# Switching to Green Lifestyles: Behavior Change of Ant Forest Users

**DOI:** 10.3390/ijerph15091819

**Published:** 2018-08-23

**Authors:** Zhaojun Yang, Xiangchun Kong, Jun Sun, Yali Zhang

**Affiliations:** 1 School of Economics and Management, Xidian University, Xi’an 710126, China; zhaojunyang@xidian.edu.cn (Z.Y.); chunkx@163.com (X.K.); 2 College of Business and Entrepreneurship, University of Texas Rio Grande Valley, Edinburg, TX 78539, USA; jun.sun@utrgv.edu; 3 School of Management, Northwestern Polytechnical University, Xi’an 710072, China

**Keywords:** persuasive system, motivation theory, Ant Forest, continuance intention, individual behavior change

## Abstract

Ant Forest is an emerging mobile application platform that engages people in environment-friendly behavior with fragmented time and helps them cultivate ecological awareness and habit. Users grow virtual trees on the platform with the energy saved from daily low-carbon activities, and Ant Forest plants real saplings in desertified areas when the “trees” become big enough. Facilitating the public’s participation in such green welfare, Ant Forest is a new-generation persuasive system with functions like social media and gamification. In addition to perceived persuasiveness in the existing literature, this study includes sense of achievement and perceived entertainment as extrinsic and intrinsic motivations, respectively, to explain people’s continuous use of such a system and consequent behavior change. The results of a survey suggest that primary task support, perceived credibility, and perceived social support associated with Ant Forest positively affect the user’s continuance intention through the mediation of perceived persuasiveness, sense of achievement, and perceiving entertaining. Furthermore, perceived persuasiveness and continuance intention lead to ultimate behavior change. The findings suggest the importance of both persuasive and motivational considerations in the implementation of new-generation persuasive systems to make them effective in the long run.

## 1. Introduction

For centuries, the rapid economic development of human societies has led to excessive consumption of natural resources and deterioration of the ecological environment from production and living activities. Humanity also faces increasingly severe environmental problems, such as global warming, deforestation, and land desertification. To address these worldwide issues, the international community is advocating “green economy”, “low-carbon economy”, and “green and low-carbon development”. Each individual’s green and low-carbon behavior contributes a little bit to environment protection, but the aggregate of such small efforts is significant.

As the largest developing country, China encourages its citizens to cultivate ecological mentality and green lifestyle by all means. Its Internet+ initiative creates opportunities for people to participate in public welfare through new mobile application platforms. Based on its Alipay platform, Ant Financial launched Ant Forest in 2016, which is a mobile application for users to manage personal carbon accounts. Featuring social interaction and game entertainment, Ant Forest promotes green welfare by encouraging individual low-carbon behavior for public benefits.

The number of Ant Forest registered users exceeded 350 million by May 2018, over 4% of the global population [1]. However, compared with Alipay’s 800 million users (as of the end of 2017), people’s involvement with Ant Forest can still be enhanced. Therefore, studying the influencing factors of the attitudes and behaviors of Ant Forest users has theoretical and practical significance for the improvement of user viscosity and the public participation in environmental protection.

## 2. Ant Forest Phenomenon

On 27 August 2016, Ant Financial announced the launch of a “carbon account” for 450 million real-name users of its Alipay platform. The official stated that this would be the world’s largest personal carbon account platform to date for measuring, trading, and sharing low-carbon lives. It allows people to record low-carbon footprints and cultivate their green lifestyle. The purpose of “carbon account” is to help people quantify and make sense of ecological accomplishment. Launched at the first phase of carbon account, Ant Forest is a mobile application that encourages people to pursue low-carbon behavior and share ecological lifestyles, and highlights their contributions to green welfare.

An individual opens an Ant Forest account and receives a virtual sapling. Then the person may record low-carbon activities, such as walking, subway/bus commuting, shared bicycle riding, offline payment, online utility payment, and online ticketing. They generate the green energy required for the growth of the sapling. In addition, the user can also “steal” green energy from friends or water their saplings. When the green energy accumulates to a certain amount, a grown-up virtual tree turns into a real sapling to be planted by a Forest Ant ecosystem partner in a needed area.

**Figure 1:**
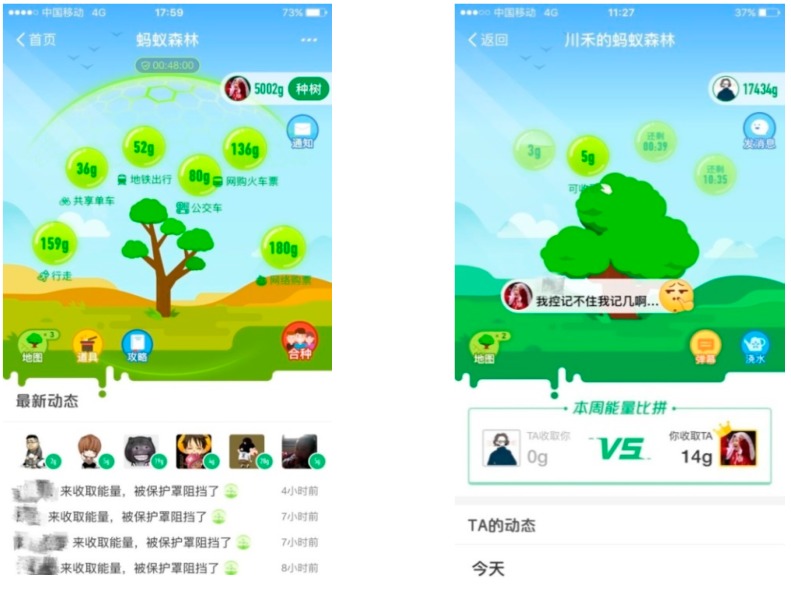
Sample Ant Forest interfaces.

An Ant Forest user’s main interface and friend interface are shown in Figure 1. The main interface includes a virtual tree, energy balls of different sources, props, and partners. The friend interface includes “stealable” energy balls, barrage, watering, and energy values collected from each other. In addition to the initial functions of “stealing” green energy from friends and watering their trees, Ant Forest later launched new “energy protection hood” and “combination” functions. Users may acquire protection hoods to prevent their friends from stealing energy, and combine individual efforts to collectively grow trees within family, lover, classmate, colleague, and friend circles. These social features enhance Ant Forest’s recreational value to users.

**Figure 2:**
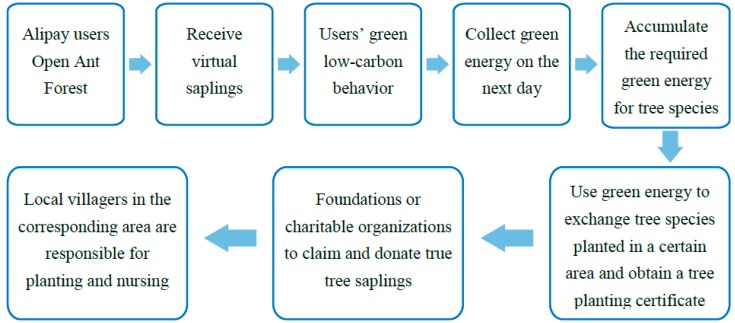
Ant Forest use process.

Figure 2 shows how a person may open an Ant Forest account, grow a virtual tree, and convert it into a real tree. Through the use of the platform, every low-carbon action can be recorded to gather green energy that eventually turns into real saplings planted in desertified areas. Ant Forest convinces people of green welfare and engages them in daily low-carbon actions and social interactions for the sense of accomplishment from fulfilling public responsibilities. In this sense, Ant Forest incorporates the design concepts of persuasive system, social networking, and game entertainment to facilitate green welfare.

By May 2018, 350 million Ant Forest users had reduced more than 2.83 million tons of carbon emission, which was converted into 55.52 million trees planted in desertified areas of about 6500 acres [1]. The concept of persuasive design of Ant Forest is recognized by United Nations Development Program (UNDP). At the Third United Nations Environment Conference held on 5 December 2017, the UNDP pointed out that Ant Forest plays a unique and significant role in the global carbon market. Erik Solheim, the executive director, believes that 200 million people around the world are practicing green lifestyle through the Ant Forest platform, which provides them instant feedback on environmental impacts and makes the process competitive and exciting. The public welfare model of Ant Forest draws the attention of many global non-governmental organizations (NGOs) as well. For instance, John Kimathi, head of the African Consultative Organization, is enthusiastic about how to let African people use Ant Forest to reduce carbon emissions [2].

User engagement in Ant Forest is completely voluntary, as it fulfills the social responsibility to participate in public environmental protection endeavor. Thus, the development of the Ant Forest platform follows the persuasive system design principle to integrate green lifestyle with environmental protection through carbon account. People get a sense of accomplishment while being entertained during the process, and are motivated to use Ant Forest continuously. Users also enjoy game-like activities and social interactions such as “stealing” energy from friends and watering their trees together with expressions and text barrage.

Therefore, people’s engagement with Ant Forest is not only influenced by the persuasive system design, but is also driven by different motivations. Yet, there is a lack of research on user behavior concerning persuasive systems similar to Ant Forest that integrates public welfare, social networking, entertainment, and environmental protection. To fill in the gap, this study uses persuasion and motivation theories to study user behavior associated with such new-generation persuasive systems. The findings may yield useful insights for the development of effective persuasive systems that people like to use continuously.

## 3. Theoretical Background

There has been a long history of studying the attitudes and behaviors of information system users. Most studies are based on utilitarian frameworks such as theory of reasoned action (TRA) [3], theory of planned behavior (TPB) [4], technology acceptance model (TAM) [5], unified theory of acceptance and use of technology (UTAUT) [6], and social cognition theory (i.e., “computer self-efficacy”) [7]. However, persuasive systems like Ant Forest are designed to change user behavior by cultivating enthusiasm and trust, not necessarily limited to utilitarian considerations. The aforementioned frameworks are somewhat insufficient to examine such behavior changes. Rather, persuasion and motivation theories are more relevant to the study of user behavior concerning persuasive systems.

### 3.1. Persuasion Theory

Faludi [8] believed that persuasion, which belongs to the field of social psychology, is a new but inaccurate science. Traditional persuasion concerns interpersonal communication that influences the autonomy of others’ judgments and actions, such as the persuasion techniques that advertisers and marketers have been using. In today’s digital age, the rapid development of information and communication technologies such as the Internet and mobile telecommunication has created new platforms and opportunities for persuasive interaction. Various systems have been developed to achieve the goal of changing the users’ attitude or behavior through their interactions with the systems.

There are two fundamental concepts in the persuasive system design: persuasion and persuasive technology. Persuasion pertains to the attempt to change people’s attitude and/or behavior without using coercion or deception, and persuasive technology is used to facilitate the process [9]. Accordingly, a persuasive system is a computer system designed to shape, change, or reinforce people’s attitude and behavior without any coercion or deception factors [10].

Fogg et al. [9] applied persuasion theory in the field of human–computer interaction, and proposed the Fogg behavioral model (FBM). The model posits that for an individual, the generation of a specific behavior must have three dimensions; namely, sufficient motivation, the ability to implement the behavior, and the triggering factor that sparks it off. This model can be used to analyze the persuasiveness of a system for design improvement.

Oinas-kukkonen and Harjumaa [11] emphasized the importance of “voluntarily” changing user behavior or attitude for persuasive systems. Successful persuasion occurs when the attitude or belief of the persuaded person changes in the direction of expectation. Oinas-Kukkonen and Shevchuk [12] proposed a persuasive system design model (PSD) that contains four major principles that must be followed in designing persuasive systems; namely, primary task support, dialogue support, system credibility, and social support. Oinas-Kukkonen and Harjumaa [13] integrated FBM and PSD into a unified framework to help design and evaluate persuasive systems.

Most of the researchers explore the factors that influence user behavior and lead to long-term behavior change by modifying and expanding the PSD model. Drozd, et al. [14] found that the primary task support and perceived credibility had positive impacts on perceived persuasiveness, which influenced actual use through behavioral intention. Lehto, et al. [15] removed social support as a design principle and studied the effect of perceived persuasiveness on intention to use and the continuance of behavior change. Based on the PSD model, Lehto and Oinas-Kukkonen [16] included social identification and sense of achievement as additional predictors of continuance intention. Alhammad and Gulliver [17] combined the PSD model with UTAUT to investigate how perceived persuasiveness is affected by perceived primary task support, perceived system credibility, and perceived social support. The study of Corbett et al. [18] found that perceived persuasiveness and integration support of a persuasive system for sustainability moderated the relationship between individual system use and environmentally responsible behavior.

### 3.2. Motivation Theory

Fogg [19] proposed motivation as one of the three behavioral factors conducive to persuasive technology design and analysis. Murphy et al. [20] showed that motivation had a significant effect on belief persuasion and behavior change. To a large extent, the long-term utilization of a persuasive system depends on user motivation. A well-designed persuasive system may keep users motivated for a longer period of time than a poorly-designed system. In this sense, motivation is an important factor that influences the use of persuasive systems.

Also called “autonomy theory”, motivation theory was first developed by Deci and Ryan [21]. According to the level of “autonomy”, motivation can be divided into three types; namely, extrinsic motivation, intrinsic motivation, and negative motivation. Among them, extrinsic motivation and intrinsic motivation have been employed to examine information systems user behavior, first in organizational settings [22] and more recently in mobile and social contexts [23,24]. Extrinsic motivation pertains to external factors such as social environment and organizational policy, whereas intrinsic motivation refers to the pure pleasure and satisfaction from using a system without being affected by outside incentives or coercion [25].

During the use of Ant Forest, users gather energy through different green activities every day, see their total energy accumulating in the friends’ rankings, and finally turn virtual trees into real ones. At the same time, users enjoy the process when they “steal” energy from each other and water trees collaboratively. Like on a social media platform, people leave funny comments and naughty barrages after they do something to friends. Therefore, there are two aspects of Ant Forest user motivation: extrinsic motivation related to environmental achievement and intrinsic motivation related to social entertainment.

## 4. Research Model

**Figure 3:**
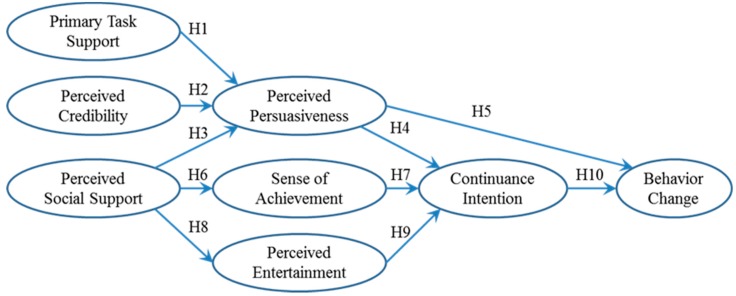
Research Model.

To examine how the use of Ant Forest cultivates people’s green lifestyle, this study combines persuasion and motivation theories with the multi-layer conceptualization of persuasive system user behavior by Lehto and Oinas-Kukkonen [16]. In the research model shown in Figure 3, the technology-facilitated behavior change process includes three stages: system experiences, cognitive antecedents, and behavioral consequences. There are multiple psychological constructs to capture each stage: system experience comprises primary tasks support, perceived credibility and perceived social support, cognitive antecedents comprise perceived persuasiveness, sense of accomplishment and perceived entertainment, and behavioral consequences comprises continuance intention and behavior change.

Primary task support refers to how well a persuasive system supports the main activities of each user [17]. Perceived credibility pertains to user trust in the services delivered, such as information, advice, and outcome [26]. Perceived social support concerns the influence from social norms and referent others (e.g., friends and family members) [16]. Together, they describe how a persuasive system shapes an individual’s experiences from the use of it. Behavioral antecedents capture the influence of system experiences and predict behavioral consequences. Perceived persuasiveness concerns an individual’s subjective evaluation of a system in terms of its impacts on his/her beliefs and values [15]. Sense of achievement refers to a series of positive inner feelings that result from an individual’s effort reaching or exceeding a certain goal, criterion, or standard [27]. Perceived entertainment pertains to the perceived level of fun or pleasure derived from using a system [28]. These three cognitive antecedents correspond to persuasion effectiveness, extrinsic motivation, and intrinsic motivation, respectively, in persuasion and motivation theories. Finally, behavioral consequences include continuance intention to keep using a persuasive system, and behavior change to consciously and intentionally switch and stick to positive behavior.

A persuasive system is designed to help users be aware of an issue, set goals, and track the progress. With Ant Forest, users save carbon emission through the daily green activities and accumulate green energy to raise virtual trees until they grow big enough to become real trees. Such primary task support is likely to have a positive impact on perceived persuasiveness [14,15].

**Hypothesis** **1.** *Primary task support positively affects perceived persuasiveness*.

For a persuasive system, its services are supposed to be reliable and trustable. In addition to the certificate that gives each real sapling a number and indicates where it is planted by whom, Ant Forest uses satellite and real-time camera technologies to let users see actual results. The purpose is to convince users of the value and importance of their persistent contributions. Therefore, perceived credibility is likely to enhance perceived persuasiveness [14,15,16,17].

**Hypothesis** **2.** *Perceived credibility positively influences perceived persuasiveness*.

People’s engagement in healthy or environment-friendly activities is closely related to social support. The design of Ant Forest allows people to add friends, “steal” their energy, and water trees for each other. Such a persuasive system motivates users’ participation by leveraging social influence. Thus, perceived social support is likely to have a positive impact on perceived persuasiveness [16,17].

**Hypothesis** **3.** *Perceived social support positively affects perceived persuasiveness*.

Perceived persuasiveness indicates the ability of a system to influence user intention. In the classical model of attitude change, information is presented, received, and processed; if the information is successfully received, recipients’ attitudes will shift toward the advocated position, and the altered attitude may have an effect on subsequent behavior [29]. Fogg et al. [9] pointed out that persuasion should lead to long-lasting attitude change without any coercion or deception. Therefore, perceived persuasiveness is likely to positively affect users’ willingness to continue using a persuasive system to shape positive behavior [14,15].

**Hypothesis** **4.** *Perceived persuasiveness positively influences continuance intention*.

**Hypothesis** **5.** *Perceived persuasiveness positively influences behavior change*.

Concerning the perceived fulfillment of the purpose for which people use a system, the sense of achievement is the main extrinsic motivation of users [30]. Under the impetus of achievement motivation, individuals will pursue and complete high-level tasks or activities and gain recognition from others. Social support is found to have a significant impact on people’s achievement, such as in educational settings [31,32]. On the Ant Forest platform, people’s sense of achievement gets enhanced when their green efforts and accomplishments (e.g., energy collected and certificates awarded) are recognized by friends.

**Hypothesis** **6.** *Perceived social support positively influences sense of achievement*.

**Hypothesis** **7.** *Sense of achievement positively influences continuance intention*.

Perceived entertainment is the intrinsic motivation that plays an important role in the acceptance and use of information technology, especially hedonic systems [33,34]. For example, the utilization of game mechanisms enhances user engagement through mutual cooperation and competition [35]. For collaborative environmental endeavors, people enjoy the social processes when they are involved in pertinent mechanisms conducive to goal attainment [36]. Ant Forest provides users gaming functions such as “stealing” friends energy, watering virtual trees for each other, leaderboard, and posting emotional barrages. Such social interactions among users bring competition and fun to repetitive activities. Therefore, perceived social support is likely to positively influence perceived entertainment, which enhances continuance [37,38].

**Hypothesis** **8.** *Perceived social support positively influences perceived entertainment*.

**Hypothesis** **9.** *Perceived entertainment positively influences continuance intention*.

Finally, altered attitude from using a persuasive system affects people’s behavior [29]. The ecological mentality induced by Ant Forest shapes new routines for people, such as offline payments, public transportations, and shared bicycles. Therefore, continuance intention is likely to have a positive influence on behavior change [39].

**Hypothesis** **10.** *Continuance intention positively affects behavior change*.

## 5. Methodology

### 5.1. Measurement

This study adapts measurement items from existing persuasive system design principle scale, achievement motivation scale, and hedonic motivation scale, and supplements them with new ones to capture Ant Forest user behavior. All the items were on a seven-point Likert scale from “strongly disagree” to “strongly agree”. Table 1 lists the items and their sources.

### 5.2. Subjects

The target population comprises Ant Forest users. An online survey elicited 303 responses and 295 were complete, leading to an effective response rate of 97%. Table 2 gives the profiles of participants in the final sample. Gender balanced, the participants were relatively young and well-educated.

## 6. Results

Common method bias (CMB) was assessed with both the traditional factor analysis method and a more recent marker-variable technique. The Harman single-factor test found that the proportion of the first principal component in the unrotated factor matrix was 42.96%. As it was below 50%, no serious common method bias was detected.

With the marker-variable technique, this study uses a theoretically irrelevant variable, fantasizing as a marker variable. Its correlations with other variables in the research model were less than 0.3. In addition, there was no change in the significance of the correlation coefficients after the partial correlation adjustments [44]. The results further dismissed the threat of CMB to statistical conclusion validity.

The main statistical tool used in this study was SmartPLS 3.2 (SmartPLS GmbH, Bönningstedt, Germany). First, measurement validity was assessed, as shown in Table 3. All the standard loadings of measurement items were above 0.7 and significant at the 0.001 level [45]. All Cronbach’s alpha and composite reliability (CR) values were greater than 0.7 [45]. The results support scale reliability.

Table 4 gives the descriptive statistics and correlation matrix to assess face validity as well as convergent and discriminant validity. Relevant to the phenomenon under investigation and related among each other in theory, all variables but the marker variable of fantasizing exhibited relatively positive responses and moderately high correlations. Convergent validity of each variable in the model was supported as the square root of average variance extracted (AVE) was greater than 0.71, indicating that AVE was above 0.5 and the majority of shared variance among measures was accounted for [45]. Meanwhile, discriminant validity was supported as the largest correlation coefficient was 0.822, lower than the smallest square root of AVE 0.871 (excluding the marker variable of Fantasizing—FA).

**Figure 4:**
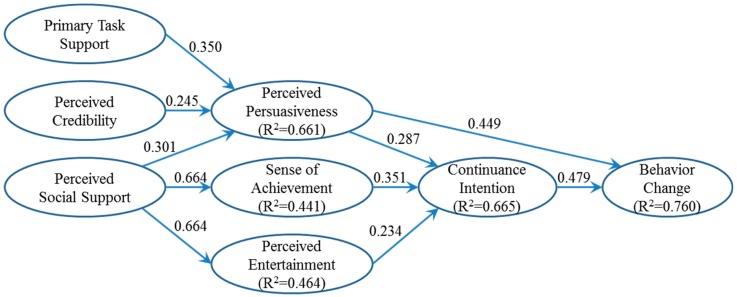
Standardized model estimates. Note: All regression weights were significant at the 0.001 level (two-tailed test).

Figure 4 reports standardized regression coefficients in the research model. All of them were positive and significant at the 0.001 level, supporting all the research hypotheses. In addition, the *R*-squared values indicated that the model explained most of the variation in behavioral consequences, including continuance intention (two-thirds) and behavior change (three-fourths). Together, system experiences account for the majority of variation in perceived persuasiveness, supporting the effectiveness of Ant Forest as a persuasive system. Perceived social support also contributed to almost half of the variation in extrinsic and intrinsic motivations, sense of achievement, and perceived entertainment, respectively. The relatively large effect sizes support the predictive power of the research model.

## 7. Discussion

In line with the persuasion theory, Ant Forest’s primary task support, perceived credibility, and perceived social support have positive impacts on perceived persuasiveness. The largest regression weight associated with primary task support shows that what convinced users the most is the fact that Ant Forest actually helps them accomplish the goal of “planting trees”. The next important factor is the social support that Ant Forest facilitates by allowing users to interact with each other. Finally, the security and reliability of Ant Forest provide the necessary guarantee of user privacy and information safety. Perceived persuasiveness then positively influences continuance intention and behavior change. The larger regression weight associated with behavior change confirms the important role that Ant Forest’s green concept plays in enhancing people’s ecological mentality.

In line with the motivation theory, sense of achievement and perceived entertainment from the use of Ant Forest enhance continuance intention. Thus, Ant Forest’s features like tree certificate, satellite image, real-time camera, and leaderboard are effective to increase user viscosity. In addition, the sense of achievement and perceived entertainment are also significantly affected by social support, with the largest regression weights (over 0.66) among all. Ant Forest’s social functions such as “stealing” energy, partnership watering, and friend interaction can indeed retain users. Its philosophy to make it easy to contribute to green welfare in a gamified way works by enhancing user sense of achievement and perceived entertainment. Meanwhile, the effect of continuance intention on behavior change is found to be no smaller than that of perceived persuasiveness. User viscosity cannot be overemphasized for persuasive systems like Ant Forest as it takes time to cultivate good habits after the attitude change from persuasion.

The findings yield helpful insights for the theory and practice concerning persuasive system implementation and usage. The main theoretical contribution is the incorporation of user motivations with the original persuasion aspect of persuasive system research [46] in the examination of behavioral change facilitated by new-generation systems like Ant Forest. Such systems include a number of features like persuasion, green welfare, social networking, and gamification, calling for a more comprehensive understanding of user behavior. Combining both persuasive and motivational routes, the research model captures users’ utilitarian, hedonic, and trust-related experiences. The validated relationships among relevant variables, therefore, provide a lens to look into such emerging user behavior.

The findings also provide useful guidance for practitioners in the design and development of new-generation persuasive systems. Rather than the traditional consideration of usability, user persuasion and user motivation are the two key success factors that lead to mentality formation and habit cultivation. By linking persuasive system design principles with user behavior research, the findings demonstrate how the persuasive and motivational aspects of system design influence continuance intention and behavior change. For persuasive systems like Ant Forest, the insights reveal the best practices on how to enhance user viscosity and promote positive behavior in a sustainable manner.

## 8. Conclusions

Based on persuasion and motivation theories, this paper proposes a behavior change model for Ant Forest, a new-generation persuasive system featuring green welfare, social networking, and gamification. The empirical findings from a user survey support hypothesized relationships. On the persuasion theory side, all major system experiences including primary task support, perceived credibility, and perceived social support make differences in perceived persuasiveness, which affects both continuance intention and behavior change. On the motivation side, perceived social support is critical for user viscosity through the mediation of sense of achievement (extrinsic motivation) and perceived entertainment (intrinsic motivation). Therefore, developers must pay attention to both persuasion and motivation in the design and implementation of persuasive systems like Ant Forest.

This study has limitations that point to the directions of future research. First, there is a lack of diversity in the sample; most participants were young people with higher education in cities. This reflects the current user base of Ant Forest as they are more open to such a new concept; yet the findings may not be generalizable to other populations, such as middle-age and senior citizens and rural citizens. It is expected that more of them will join the Ant Forest community in the next few years. Future studies may include them in the samples, and compare the results with those obtained from early adopters. Similarly, observations can be collected from outside of China after Ant Forest and similar systems are used by other developing countries in which people have different levels of environmental awareness [47].

## Figures and Tables

**Table 1:** Measurement items and sources.

Variable	Item	Source
Primary Task Support	The AF helps me in reaching my goals gradually.	[16]
	The AF helps me change my green life/behavior habits.	[15]
	With AF, I can plant a tree through the hard work.	Self-developed
	AF allows me to participate in environmental protection.	Self-developed
Perceived Credibility	In my opinion, the AF is trustworthy.	[16]
	In my opinion, the AF is reliable/believable.	[16]
	I believe AF will plant a real tree when my virtual tree matures.	Self-developed
	AF can be trusted in user privacy protection.	Self-developed
Perceived Social Support	People who influence my behavior think that I should use AF.	[6]
	People who are important to me think that I should use AF.	[6]
	Using AF is a trend, which I have to keep up with.	Self-developed
	Many people around me are using AF and I want to become a member.	Self-developed
Perceived Persuasiveness	AF has an influence on me.	[15]
	AF is personally relevant for me.	[15]
	AF makes me reconsider my green life habits.	[15]
	AF makes me want to increase my green activity.	[40]
	I think AF would be useful in increasing my green activity.	[40]
	I appreciate what AF is designed to do.	Self-developed
Sense of Achievement	When I “planted” a tree, I was very excited.	[41]
	When I “planted” a tree, I felt a sense of achievement.	Self-developed
	When I “planted” a tree, I was very proud.	[41]
	I am very satisfied with what I have done with AF.	[41]
	I think it’s worth the time and effort to use AF.	[41]
Perceived Entertainment	Using AF is fun.	[6]
	Using AF is enjoyable.	[6]
	Using AF is very entertaining.	[6]
Continuance Intention	I will continue to use AF.	[6]
	I will keep using AF frequently	[6]
	I would like to introduce AF to my relatives and friends.	[6]
Individual Behavior Change	Since my enrollment, I have developed a daily routine to use AF.	[42]
	Since I used AF, my daily life has become greener and healthier.	[42]
	Whenever there is a chance, I take an action that can produce AF green energy.	[42]
Fantasizing (as a marker variable)	I daydream a lot.	[43]
	When I go to the movies I find it easy to lose myself in the film.	[43]
	I often think of what might have been.	[43]

Note: AF—Ant Forest.

**Table 2:** Sample characteristics.

Variable	Options	Frequency	Proportion
Gender	Males	150	50.8%
	Females	145	49.2%
Age	18–25	227	76.9%
	26–30	58	19.7%
	31–40	10	3.4%
Education	Elementary	5	1.7%
	High-school	13	4.4%
	Undergraduate	120	40.7%
	Master	141	47.8%
	Ph.D.	8	2.7%
	Missing/Unreported	8	2.7%

**Table 3:** Reliability and Composite Reliability (CR).

Variable	Items	Loadings	Cronbach’s α	CR
Primary Task Support	PTS1	0.902	0.894	0.926
	PTS2	0.863		
	PTS3	0.813		
	PTS4	0.904		
Perceived Credibility	PC1	0.929	0.923	0.946
	PC2	0.948		
	PC3	0.885		
	PC4	0.844		
Perceived Social Support	SS1	0.873	0.895	0.927
	SS2	0.865		
	SS3	0.874		
	SS4	0.876		
Perceived Persuasiveness	PP1	0.897	0.950	0.962
	PP2	0.897		
	PP3	0.939		
	PP4	0.938		
	PP5	0.926		
	PP6	0.802		
Sense of Achievement	SA1	0.927	0.953	0.963
	SA2	0.933		
	SA3	0.934		
	SA4	0.908		
	SA5	0.861		
Perceived Entertainment	PE1	0.956	0.959	0.974
	PE2	0.970		
	PE3	0.959		
Continuance Intention	CU1	0.927	0.895	0.935
	CU2	0.903		
	CU3	0.912		
Individual Behavior Change	BC1	0.908	0.901	0.938
	BC2	0.933		
	BC3	0.886		
Fantasizing	FA1	0.913	0.801	0.882
	FA2	0.902		
	FA3	0.706		

**Table 4:** Descriptive statistics and correlation matrix.

	Mean	PTS	PC	SS	SA	PE	PP	CU	BC	FA
PTS	5.288 (1.285)	**0.871**								
PC	5.470 (1.239)	0.797	**0.903**							
SS	4.812 (1.360)	0.714	0.679	**0.872**						
SA	5.595 (1.197)	0.768	0.742	0.664	**0.913**					
PE	5.382 (1.372)	0.754	0.692	0.664	0.815	**0.962**				
PP	5.136 (1.255)	0.761	0.729	0.718	0.816	0.818	**0.901**			
CU	5.403 (1.304)	0.744	0.655	0.637	0.776	0.754	0.765	**0.909**		
BC	4.999 (1.464)	0.728	0.645	0.666	0.734	0.747	0.815	0.822	**0.914**	
FA	4.059 (1.465)	0.151	0.138	0.242	0.145	0.176	0.247	0.122	0.224	**0.846**

Note: Standard deviations are included in the parentheses beside the means. Bolded on the diagonal of correlation matrix are the square roots of average variance extracted (AVEs). PTS—primary task support; PC—perceived credibility; SS—perceived social support; SA—sense of achievement; PE—perceived entertainment; PP—perceived persuasiveness; CU—continuance intention; BC—individual behavior change; FA—fantasizing (marker variable).
